# Protein losses and nitrogen balance during continuous renal replacement therapy

**DOI:** 10.1186/cc9801

**Published:** 2011-03-11

**Authors:** H Hayami, O Yamaguchi, M Shimosaka, H Fujimoto, S Tsuboi, M Satou

**Affiliations:** 1 Yokohama City University Medical Center, Yokohama, Japan

## Introduction

Acute renal failure (ARF) is a highly catabolic state and mean normalized catabolic rates of 1.5 g/kg/day protein have been reported. In hemodynamically unstable ARF patients, continuous renal replacement therapy (CRRT) has become a popular treatment modality, but may have the disadvantage of producing substantial protein losses, reported to be as high as 1.3 g/l. In the USA and Europe, CRRT outputs reach 50 l/day, and this value would amount to protein losses of up to 65 g/day. ASPEN and ESPEN guidelines recommend that these patients should receive increased protein, up to a maximum of 2.5 g/kg/day, and that protein should not be restricted in patients with ARF as a means to avoid or delay initiation of dialysis therapy. But most previous studies were conducted in the era when energy requirements were adjusted by stress factors, and without intense glucose control therapy. So the optimal amount of protein supplementation in ARF patients in recent nutritional control is still unknown. In Japan, due to the limitation of doses of dialysate by health insurance it remains only 15 l/day, and protein losses are expected to be smaller than western countries. We measured the amount of nitrogen concentration in dialysate/ultrafiltrate samples, and calculated the nitrogen balance in such patients.

## Methods

We analysed eight critically ill patients requiring CRRT in the ICU in a university hospital retrospectively. Patients received NPC 25 kcal/kg/day increasing to the target over the next 2 to 3 days, preferably by enteral (postpyloric) route if possible. The dose of protein intake differed mainly due to BUN concentration (70 mg/dl was acceptable).

## Results

Of eight patients, six died (D) and two survived (S). Days of CRRT treatment were 11.7 ± 5.2 (4 to 20) in group D versus 9.0 ± 5.7 (5 to 13) in group S, and 24 hours creatinine clearance of CRRT was 9.6 ± 2.9 versus 10.5 ± 3.6 ml/minute/m^2^, dialysate/ultrafiltrate nitrogen loss was 6.4 ± 3.3 versus 8.5 ± 4.1 g/day, and nitrogen balance was -0.08 ± 0.48 versus -0.034 ± 0.44 g/kg/day (-5.7 ± 6.6 vs. 2.6 ± 6.1 g/day), retrospectively. The estimated amount of protein loss was expected to be almost 40 g/day.

## Conclusions

Nitrogen losses in dialysate/ultrafiltrate samples were larger than previously reported even in a smaller dialysate/ultrafiltrate dose. In ICU patients with ARF, protein requirements can differ and have to be assessed individually. Large, prospective, randomized, controlled studies are needed to optimize the dosing of protein in critically ill patients with ARF who are treated with CRRT and the effects on patient morbidity and mortality.

